# Knockdown of LncRNA PANDAR by CRISPR-dCas9 Decreases Proliferation and Increases Apoptosis in Oral Squamous Cell Carcinoma

**DOI:** 10.3389/fmolb.2021.653787

**Published:** 2021-03-26

**Authors:** Tingting Jia, Fengze Wang, Bo Qiao, Yipeng Ren, Lejun Xing, Haizhong Zhang, Hongbo Li

**Affiliations:** ^1^ Department of Stomatology,The First Medical Center of Chinese PLA General Hospital, Beijing, China; ^2^ Clinic of Oral and Cranio-Maxillofacial Surgery, University Hospital Basel, Basel, Switzerland

**Keywords:** CRISPR-dCas9, oral squamous cell carcinoma, PANDAR, SRFS7, PIM1

## Abstract

Oral squamous cell carcinoma (OSCC) is the most common malignant epithelial tumor in the oral cavity. Emerging evidence has demonstrated the important function roles of long noncoding RNAs (lncRNAs) in human cancers. LncRNA promoter of CDKN1A antisense DNA damage activated RNA (PANDAR) functions as an oncogene in multiple carcinomas, whereas its function in OSCC has not been investigated yet. The aim of our study is to investigate the possible regulatory mechanism of PANDAR in OSCC. First of all, PANDAR was highly expressed in OSCC cells and loss-of-function assays mediated by CRISPR-dCas9 observed that PANDAR silencing restrained cell proliferation and promoted cell apoptosis. Then we found and confirmed the interaction between PANDAR and serine and arginine rich splicing factor 7 (SRSF7). Subsequently, serine/threonine-protein kinase pim-1 (PIM1) was proved to be regulated by PANDAR in SRSF7-dependant way. Rescue experiments validated that PANDAR modulated the proliferation and apoptosis in OSCC through PIM1. In conclusion, PANDAR bound with SRSF7 to increase PIM1 expression, hence promoting the development of OSCC. These data shed new lights into the seeking for effective diagnostic biomarkers and therapeutic targets for OSCC patients.

## Introduction

Oral squamous cell carcinoma (OSCC) is a well-known cancer that accounts for more than 90% of all types of oral cancers (Bagan et al., 2010). Due to efforts made on cancer therapy, such as radiotherapy, chemotherapy, and molecular target therapy, the 5-year relative survival more than doubled in the last 26 years for OSCC (Sutton et al., 2003; Jerjes et al., 2010; van Putten et al., 2018). Nevertheless, the pathogenesis and molecular mechanism of OSCC have not been clear, and further research is needed.

Long noncoding RNAs (lncRNAs) are noted as nonprotein coding transcripts which are longer than 200 nucleotides (nt) in length (Xing et al., 2016). Over the past years, researchers have identified thousands of lncRNAs and also demonstrated their roles in physiological and pathological processes of diseases (Du et al., 2018; Ye et al., 2018). For example, SP1-induced lncRNA CASC11 promotes the tumorigenesis of glioma by targeting FOXK1 through sponging miR-498 (Jin et al., 2019). APF lncRNA affects autophagy and myocardial infarction by sponging miR-188-3p (Wang et al., 2015). DLX6-AS1 promotes pancreatic cancer development by regulating miR-497-5p/FZD4/FZD6/Wntβ-catenin pathway (Yang et al., 2019). Although lncRNAs have been widely studied in many diseases, more investigations about the aberrant lncRNAs in OSCC are urgently needed.

LncRNA promoter of CDKN1A antisense DNA damage activated RNA (PANDAR), which is located in chromosome 6p21.2, functions an oncogene in many kinds of tumors, such as pancreatic ductal adenocarcinoma (Jiang et al., 2017), breast cancer (Li et al., 2019), gastric cancer (Liu et al., 2018), acute myeloid leukemia (Yang et al., 2018), retinoblastoma (Sheng et al., 2018), ovarian cancer (Wang et al., 2018), and colorectal and cervical cancer (Huang et al., 2017; Rivandi et al., 2019). However, the regulation mechanism of PANDAR in OSCC is to be investigated.

Besides for lncRNAs, the functions of serine/threonine-protein kinase pim-1 (PIM1) in multiple cancers have already been reported by researches. Alpinumisoflavone promotes apoptosis in esophageal squamous cell carcinoma via modulation of miR-370/PIM1 signaling (Han et al., 2016). MicroRNA-33a silencing increases cyclin-dependent kinase 6, cyclin D1, and PIM1 expression and promotes cell proliferation in gastric cancer (Wang et al., 2015). Epstein-Barr virus-encoded LMP1 regulates Pim1 kinase expression to promote proliferation of cancer cells (Ding et al., 2019). Physcion 8-O-β-glucopyranoside represses tumor growth of hepatocellular carcinoma via downregulation of PIM1 (Wang et al., 2017). The investigation for the relationship between lncRNAs and PIM1 in OSCC is vitally imperative.

In this study, we aim to reveal novel regulatory mechanisms of PANDAR in OSCC. First of all, we found that PANDAR was highly expressed in OSCC cells. It was observed from CRISPR-mediated functional assays that PANDAR silencing restrained cell proliferation and promoted cell apoptosis in OSCC. Then we found and confirmed the binding between PANDAR and SRSF7. Next, PIM1 was revealed to be regulated by PANDAR through SRSF7 inhibition. Rescue experiments validated that PANDAR modulated the proliferation and apoptosis in OSCC through PIM1.

## Materials and Methods

### Cell Culture

A human immortalized oral keratinocytes (NOKs) and the five most commonly used OSCC cell lines (SAS, Cal27, SCC9, SCC15, and SCC4) from the Institute of Biochemistry and Cell Biology at the Chinese Academy of Sciences (Shanghai, China) were used in this study. All cell lines were cultured in RPMI 1640 (Gibco, Darmstadt, Germany) supplemented with 10% fetal bovine serum (FBS; Gibco), 100 U/ml penicillin, and 100 mg/ml streptomycin (Invitrogen, Carlsbad, CA) at 37°C in 5% CO_2_.

### Cell Transfection

At 24 h prior to transfection, cells were cultured in 6-well plates. Then, the cells were transfected using Lipofectamine 2000 (Thermo Fisher Scientific, Inc., Waltham, MA, United States). All cells were harvested after 48 h. Single guide RNAs (sgRNAs) targeting PANDAR and SRSF7 (sgRNA-PANDAR#1/2/3 and sgRNA-SRSF7) and the negative control sgRNA (sgRNA-NC) were constructed by RiboBio (Guangzhou, China). The sequences of sgRNAs targeting PANDAR are as follows: sgRNA-PANDAR#1GGCCAGACCTATAATATTAA; sgRNA-PANDAR#2GCCAGACCTATAATATTAAT; sgRNA-PANDAR#3GGAGATACCACCACTGTCAA. The overexpression of PIM1 was achieved by treating cells with pcDNA3.1/PIM1 and empty control (RiboBio).

### RNA Extraction and Quantitative Real-Time PCR Assays

According to the recommendations provided by manufacturer, total RNA was extracted from cells using TRIzol reagent (Invitrogen). Then the PrimeScript reverse transcriptase reagent kit (Takara Bio Inc., Kusatsu, Shiga, Japan) was used to synthesize complementary DNA (cDNA). Real-time PCR amplification was then performed using an SYBR Green Real-Time PCR Kit on the Bio-Rad CFX96 System (Applied Biosystems, Foster City, CA). Furthermore, GADPH was used as the endogenous control. The 2^−ΔΔCT^ method was used for transcript quantification. The primers for genes are as follows: PANDAR forward 5′-CTC​CAT​CAT​GCC​AAG​TTC​TGC-3′ and reverse 5′-GAA​GGC​AGG​CAA​GAC​TCG​AA-3′; SRSF7 forward 3′-GCG​GTA​CGG​AGG​AGA​AAC-5′ and reverse 3′-TCG​GGA​GCC​ACA​AAT​CAC-5′; Pim1 forward 5′-CTT​CGG​CTC​GGT​CTA​CTC​AG-3′, reverse 5′-AGT​GCC​ATT​AGG​CAG​CTC​TC-3′; GAPDH forward 5′- TGC​ACC​ACC​AAC​TGC​TTA​GC -3′, and reverse 5′- GGC​ATG​GAC​TGT​GGT​CAT​GAG -3′.

### CCK-8 Assays

The proliferation rate of cells was determined via Cell Counting Kit-8 (CCK-8) assay kit. The cells were seeded in 96-well plates (3 × 10^3^ cells/well) and cultured for five periods (0, 24, 48, 72, and 96 h) at 37°C with 5% CO_2_. At the same time, each well was added with 10 µL of CCK-8 solution and incubated for 4 h continuously. Finally, the OD value (450 nm) was assessed with a Bio-Rad iMark microplate absorbance reader (Bio-Rad Laboratories Inc., Hercules, CA, United States).

### RNA Immunoprecipitation Assay

The EZ-Magna RIP RNA-Binding Protein Immunoprecipitation kit (Millipore, Bedford, Massachusetts, United States) was applied to perform RIP assay. A total of 2 × 10^7^ cells were harvested and lysed in 100 µl lysis buffer (Millipore) for RIP reaction. Anti-IgG or anti-SRSF7 was added into cell lysates, and then the whole-cell extract was incubated with rotation overnight at 4°C. Finally, the immunoprecipitated RNA was purified using TRIzol regent, and binding targets were analyzed with qRT-PCR.

### EdU Assays

To examine cellular proliferation, 5′-Ethynyl-2′-deoxyuridine (EdU; RiboBio, Guangzhou, China) incorporation experiment was performed in light of the operational manual. When cell confluence is up to 80%–90%, all cells were incubated by EdU diluent for 2 h at 37°C. Subsequently, cells were fixed and then stained with Apollo 567 working solution for 30 min away from light. After staining, the cells were washed by penetrant. Images were captured and photographed under a fluorescence microscope (Leica, Wetzlar, Germany).

### Biotinylated RNA Pull-Down Assays

Chemically synthesized probes for PANDAR-WT, PANDAR-MUT, PIM1-WT, PIM1-MUT, and their relative negative control (NC) were biotin-labeled (they were named as Bio-PANDAR-WT, Bio-PANDAR-MUT, Bio-PIM1-WT, Bio-PIM1-MUT, and Bio-NC) using the Biotin RNA Labeling Mix (Roche Diagnostics, Indianapolis, IN). The biotinylated RNA was incubated overnight with lysates from cells and then treated with magnetic beads with *Streptomyces* for 48 h. Finally, the RNA present in the pull-down material was assessed using qRT-PCR.

### Western Blotting Assay

By using RIPA reagent (Beyotime, Beijing, China) and protease inhibitor cocktail, proteins were separated by 10% SDS-PAGE and transferred to PVDF membranes. The membranes were blocked with 5% nonfat milk for 60 min and incubated with primary antibodies at 4°C for 12–16 h. Autoradiograms were monitored by densitometry through Quantity One software (Bio-Rad). Antibodies for PIM1 and GAPDH were obtained from Cell Signaling Technology (Danvers, MA, United States).

### Caspase-3 Activity Assays

The activity of Caspase-3 was determined at 48 h after transfection by Caspase-3 activity kit (Beyotime Institute of Biotechnology, Guangzhou, Guangdong, China). Protein samples were obtained through lysis buffer and then diluted to 50 µl of final volume. The diluted protein was subjected to 75 µl of caspase-3 substrate for 3 h, and the hydrolysis of Ac-DEVD-pNA was detected at 405 nm by caspase-3 released free pNA (yellow formazan product).

### Statistical Analysis

All the numerical data are presented as the means ± SD. Each experimental procedure was repeated for more than two times. Student’s *t*-test and one-way ANOVA were used to analyze significant difference of groups, and *p* < 0.05 was taken as the significance threshold. Statistical analyses were performed with SPSS 13.0 Software (GraphPad Software, San Diego, CA, United States).

## Results

### PANDAR Was Highly Expressed in OSCC and PANDAR Inhibition Restrained Cell Proliferation and Promoted Cell Apoptosis

**FIGURE 1:**
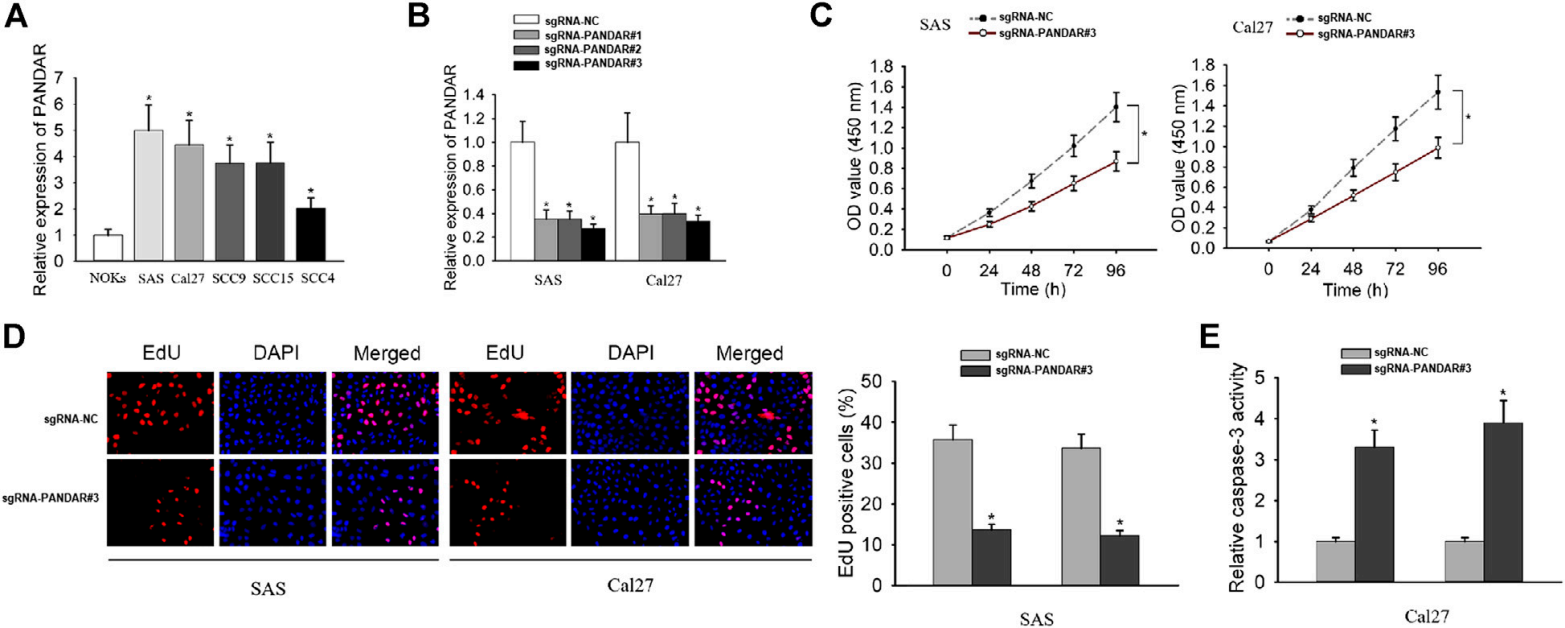
The impact of PANDAR knockdown on proliferation and apoptosis of OSCC cells. (**A)** The relative expression level of PANDAR in cancerous cell lines and normal cell line. (**B)** The knockdown efficiency of specific sgRNAs targeting PANDAR was detected in OSCC cells by qRT-PCR. (**C–E)** Cell proliferation assays (CCK-8 and EdU) and cell apoptosis assay (caspase-3 activity assay) were carried out to assess the functional role of PANDAR knockdown in OSCC. Data were represented as the mean values (±SD) of three independent experiments. ^*^ *p* < 0.05, significantly different from the control.

To explore whether lncRNA PANDAR affects the biological activities of OSCC cells, we initially examined the expression level of PANDAR in OSCC cells (SAS, Cal27, SCC9, SCC15, and SCC4) and normal oral keratinocytes (NOKs). The high levels of PANDAR in OSCC cells were shown in Figure 1A. PANDAR was accordingly silenced by transfecting with sgRNA-PANDAR#1/2/3 in SAS and Cal27 cells and sgRNA#3 achieved the best performance (Figure 1B). Therefore, this sgRNA was chosen for the following functional studies. The analysis from CCK-8 assay found that cell proliferation was inhibited after PANDAR silencing (Figure 1C). Also, the repression on cell proliferation was observed when PANDAR was knocked down, as tested by EdU assay (Figure 1D). Caspase-3 activity assay revealed that PANDAR downregulation had a stimulative effect on cell apoptosis (Figure 1E). In order to further prevent the false positive problem caused by potential off-targeting effects of sgRNA, we also used sgRNA # 1 and 2 to repeat the above functional experiments and obtained similar results (Supplementary Figure S1). These findings illustrated that silencing of PANDAR inhibited cell proliferation and induced cell apoptosis.

### PANDAR Bound With SRSF7

**FIGURE 2:**
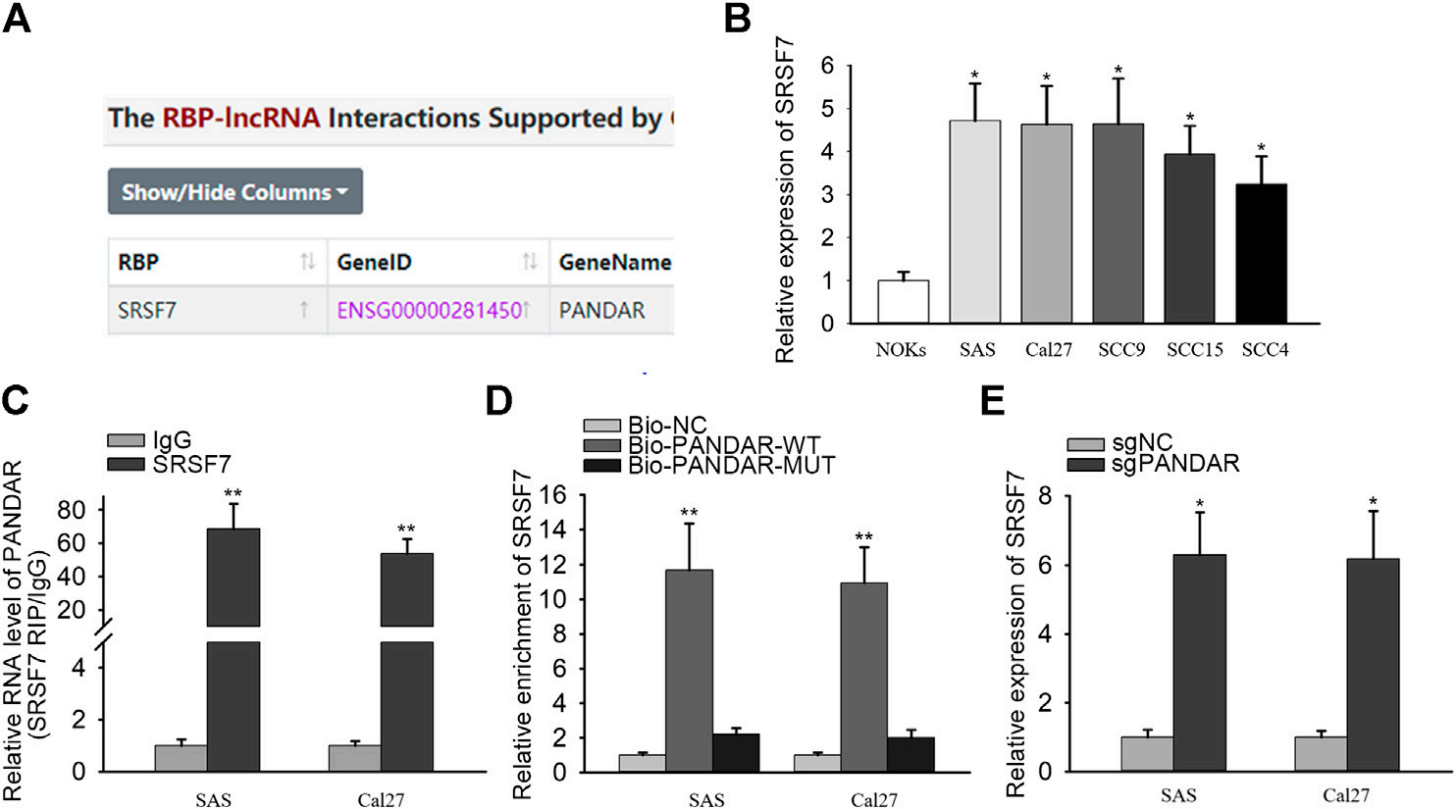
SRSF7 acted as a RBP of PANDAR in OSCC. (**A)** In line with the prediction of starBase, SRSF7 was found to be a RBP of PANDAR. **(B)** The expression level of SRSF7 in cancerous cells and normal cells. (**C,D)** RIP and RNA pull-down assays were conducted for validating the interaction between SRSF7 and PANDAR in OSCC cells. (**E)** PANDAR knockdown increased SRSF7 expression in OSCC cells. Data were represented as the mean values (±SD) of three independent experiments. ^*^ *p* < 0.05 and ^**^ *p* < 0.01, significantly different from the control.

LncRNAs have been reported to bind with RNA-binding proteins (RBPs) so as to influence cellular processes (Nguyen et al., 2018; Zhang et al., 2018). From starBase v3.0, the binding between PANDAR and SRSF7 was uncovered (Figure 2A). SRSF7 expression was upregulated in OSCC cells compared to NOKs cells (Figure 2B). Then RIP and RNA pull-down assays were performed and the results demonstrated that PANDAR was only enriched in anti-SRSF7 group and merely bio-PANDAR-WT pulled down SRSF7 protein, suggesting the direct interaction between PANDAR and SRSF7 (Figures 2C,D). Moreover, after knocking down of PANDAR, the SRSF7 expression was increased. These results indicated that PANDAR bound with SRSF7 and downregulated SRSF7.

### PANDAR Regulated PIM1 Expression Through Binding With SRSF7

**FIGURE 3:**
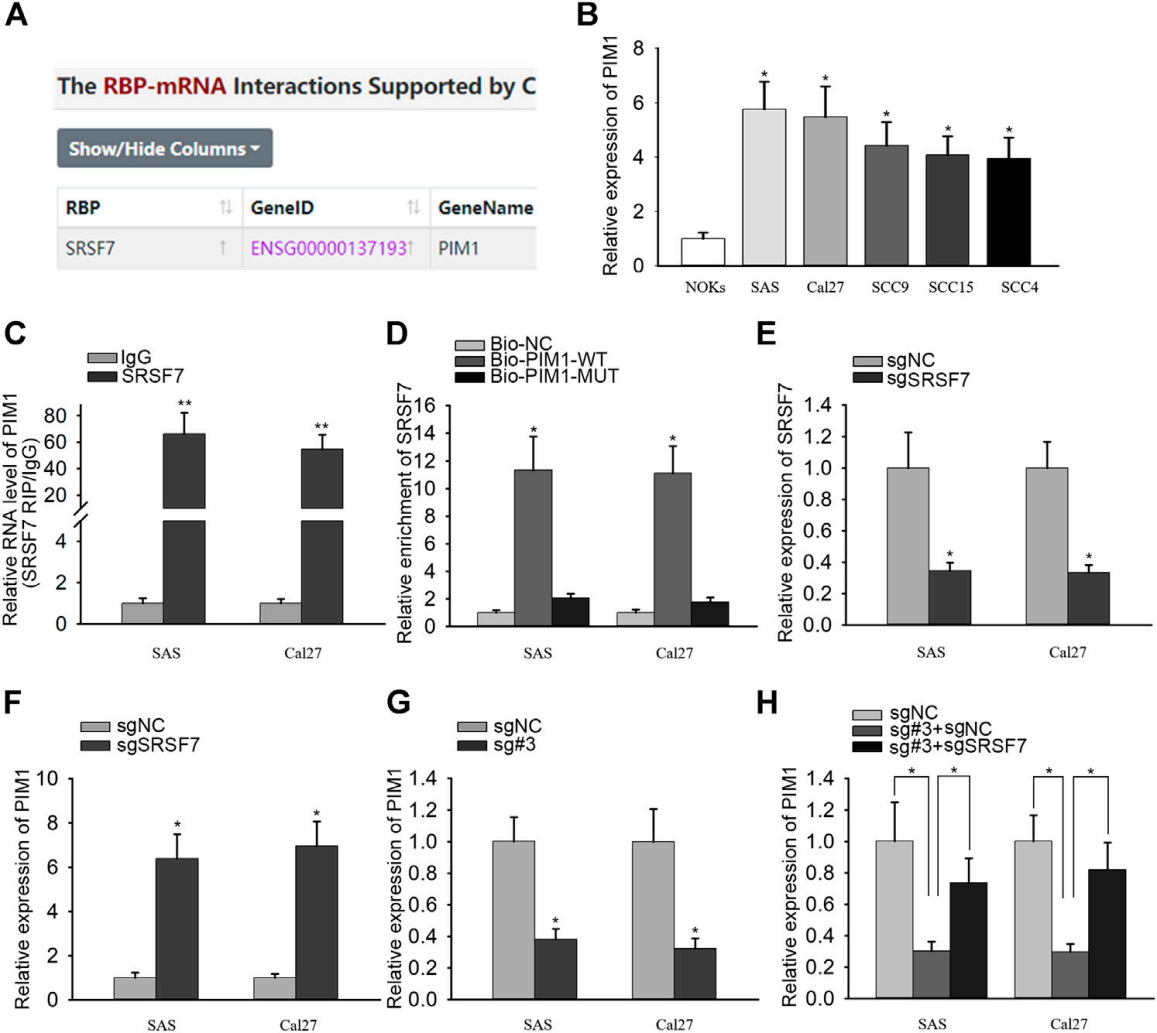
PANDAR affected PIM1 expression by regulating SRSF7 in OSCC. (**A)** According to starBase, SRSF7 was identified as a RBP of PIM1. (**B)**. The high expression level of PIM1 in OSCC cells. (**C,D)** The interaction between SRSF7 and PIM1 was confirmed by RIP and RNA pull-down assays. (**E,F)** The sgRNA targeting SRSF7 decreased SRSF7 and increased PIM1 expression. (**G,H)** PIM1 expression level was reduced by sgRNA PANDAR#3, and this effect was reversed partially by sgRNA SRSF7 in OSCC cells. Data were represented as the mean values (±SD) of three independent experiments. ^*^ *p* < 0.05 and ^**^ *p* < 0.01, significantly different from the control.

RBP interactions with mRNAs are identified in numerous cancers (Chand et al., 2017; Zhong et al., 2019). PIM1 is an oncogene in OSCC and has been known to regulate the proliferation ability of OSCC cells. Strikingly, we found out the interaction between SRSF7 and PIM1 from starBase v3.0 (Figure 3A). qRT-PCR analysis detected that PIM1 was highly expressed in OSCC cells (Figure 3B). The binding between SRSF7 and PIM1 was also confirmed through RIP and RNA pull-down experiments. In RIP assay, the enrichment of PIM1 was observed in SRSF7 group (Figure 3C). In RNA pull-down assay, SRSF7 was simply pulled down by bio-PIM1-WT probe (Figure 3D). In addition, PIM1 expression was augmented after SRSF7 was downregulated or PANDAR was knocked down (Figures 3E–G). The coinfluences of PANDAR and SRSF7 on PIM1 were measured through qRT-PCR. PIM1 expression was silenced by PANDAR inhibition but recovered partly by SRSF7 downregulation (Figure 3H). Taken together, PANDAR modulated PIM1 through binding to SRSF7.

### PANDAR Affected the Proliferation and Apoptosis in OSCC Through PIM1

**FIGURE 4:**
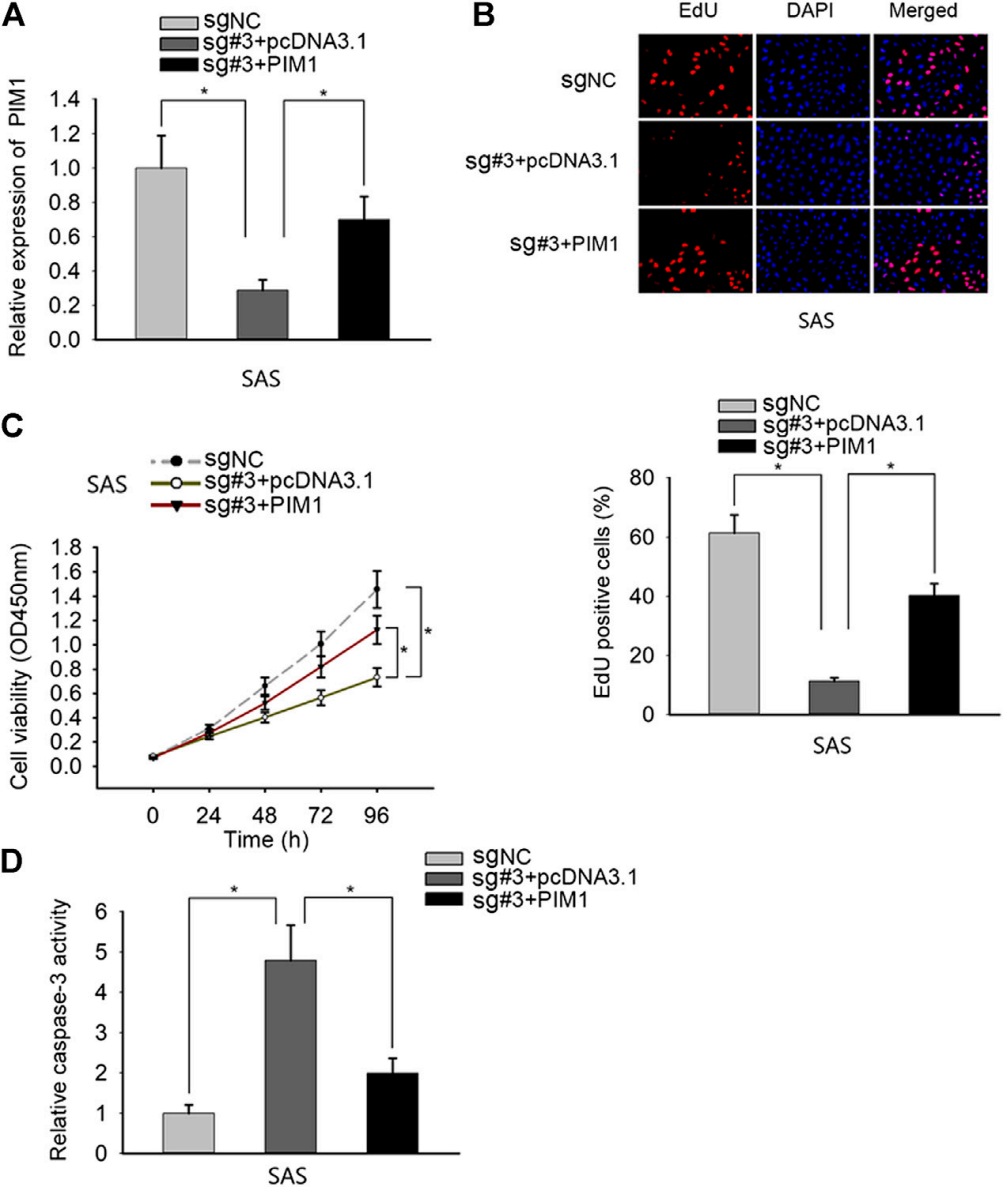
PANDAR modulated OSCC cell proliferation and apoptosis via SRSF7/PIM1 axis. (**A)** The pcDNA3.1/PIM1 reversed the inhibitory effect on PIM1 mediated by knockdown of PANDAR. (**B–D)** The inhibited proliferation and increased apoptosis of OSCC cells caused by PANDAR knockdown were recovered and weakened through overexpressing PIM1. Data were represented as the mean values (±SD) of three independent experiments. ^*^ *p* < 0.05, significantly different from the control.

Finally, we performed rescue experiments to validate the whole mechanism in OSCC. SAS cells were transfected with sgRNA-NC, sgRNA#3 + pcDNA3.1, and sgRNA#3 + PIM1, respectively (Figure 4A). Through EdU and CCK-8 assays, we observed that the proliferation ability repressed by PANDAR knockdown was rescued by PIM1 overexpression (Figures 4B,C). In caspase-3 activity assay, PANDAR repression activated cell apoptosis, and this phenomenon was counteracted when PIM1 was upregulated (Figure 4D). All in all, overexpression of PIM1 reversed the corresponding effects of PANDAR inhibition on cell proliferation and apoptosis.

## Discussion

Numerous researches have demonstrated the crucial roles of long noncoding RNAs (lncRNAs) in human tumors, including OSCC. However, further explorations are needed.

In this study, we investigated the lncRNA PANDAR in OSCC. We showed that PANDAR expression is upregulated in OSCC cells in comparison with normal oral keratinocytes, and knockdown of PANDAR by CRISPR-dCas9 reduces proliferation and increases apoptosis of OSCC cells. We also demonstrated that those phenotypes are dependent of PIM1 regulation, via binding to SRSF7.

First of all, we investigated the expression pattern and biological function of PANDAR in OSCC. Mounting evidence has illustrated the oncogenic function of PANDAR in other carcinomas except for OSCC. For instances, PANDAR promotes cell proliferation and suppresses cell apoptosis in pancreatic ductal adenocarcinoma (Jiang et al., 2017); inhibition of PANDAR reduces cell proliferation and cell invasion and suppresses EMT process in breast cancer (Li et al., 2019); PANDAR blocks CDKN1A gene transcription via competitive interaction with p53 protein in gastric cancer (Liu et al., 2018); SP1-induced PANDAR regulates cell growth and apoptosis of retinoblastoma cells (Sheng et al., 2018). Our research firstly gives the evidence for the high expression of PANDAR in OSCC cells. Loss-of-function experiments suggest that inhibition of PANDAR restrained the proliferation capacity of OSCC cells.

RNA-binding proteins (RBPs) provide one connector through which lncRNAs regulate mRNAs expression specifically (Glisovic et al., 2008; Barbagallo et al., 2018). According to the data from starBase v3.0, PANDAR was merely predicted to interact with SRSF7. In the past studies, SRSF7 has been revealed to be heightened in carcinomas and promotes cancer progression in various tumors. For example, inhibition of SRSF7 promotes apoptosis in colon and lung cancers (Boguslawska et al., 2016). MicroRNAs modulate the expression of osteopontin splice variants in renal cancer cells by targeting SRSF7 splicing factor (Boguslawska et al., 2016). The comparative expression patterns and diagnostic efficiencies of HNRNPA1 and SR splicing factors were discovered in gastric and colorectal cancers (Park et al., 2016). The present research discovered the interaction between PANDAR and SRSF7. Additionally, increasing reports have indicated that PIM1 is a tumor growth promoter via influencing cell proliferation and apoptosis (Wang et al., 2015; Wang et al., 2017; Ding et al., 2019), which forced us to study the potent relationship among the three genes. Interestingly, the binding between SRSF7 and PIM1 was unveiled though the assistance of starBase v3.0 website. Further investigations validated the regulatory role of PANDAR/SRSF7/PIM1 axis in OSCC, where PANDAR modulated the proliferation and apoptosis of OSCC through PIM1. Therefore, PANDAR/SRSF7/PIM1 axis may be a potential therapeutic target for the treatment of OSCC.

This study also highlights the important role of CRISPR-dCas9 in lncRNA research. Through the flexible regulation of lncRNA expression level, it would be easy to study the function and molecular regulation mechanism of lncRNAs (Dominguez et al., 2016; Pulecio et al., 2017; Gjaltema and Schulz, 2018). This is an unprecedented convenience.

In summary, in OSCC cells, PANDAR interacts with SRSF7 to upregulate PIM1 expression, therefore promoting the proliferation ability of OSCC cells. These data provided novel insights into the seeking for effective biomarkers and therapeutic targets for patients with OSCC.

## Data Availability

The original contributions presented in the study are included in the article/Supplementary Material; further inquiries can be directed to the corresponding authors.
