# Supplementary figures and images for: Knockdown of LncRNA PANDAR by CRISPR-dCas9 Decreases Proliferation and Increases Apoptosis in Oral Squamous Cell Carcinoma
Source: Front Mol Biosci. 2021 Mar 26;8:653787. doi: 10.3389/fmolb.2021.653787 (PMC8032867; doi:10.3389/fmolb.2021.653787)

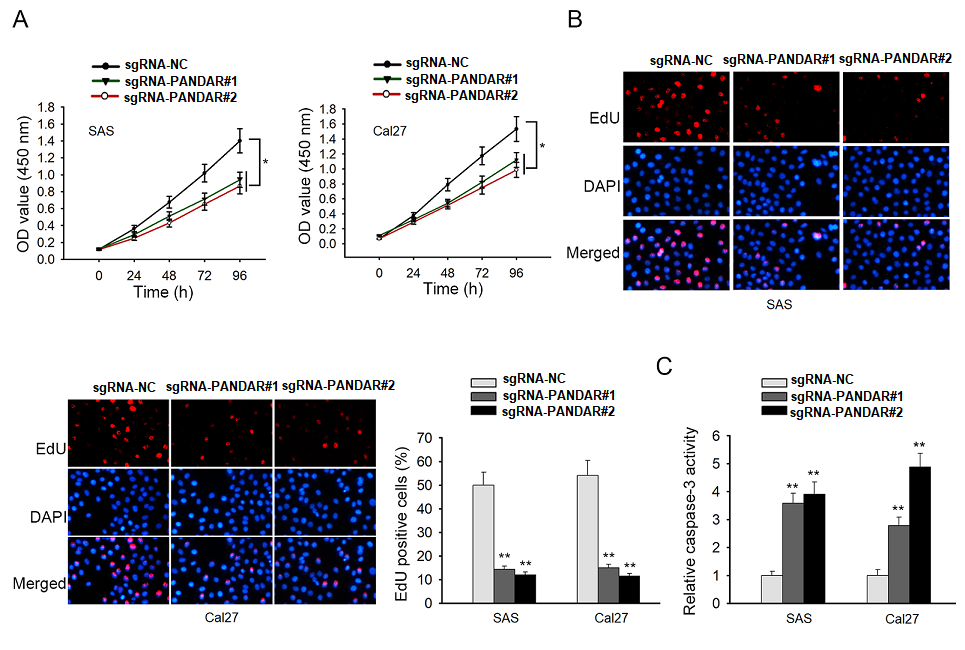

Supplement: Supplementary file 1 [file image1.tif]
